# Low-Cost Dynamometer for Measuring and Regulating Wrist Extension and Flexion Motor Tasks in Electroencephalography Experiments

**DOI:** 10.3390/s24175801

**Published:** 2024-09-06

**Authors:** Abdul-Khaaliq Mohamed, Muhammed Aswat, Vered Aharonson

**Affiliations:** 1 School of Electrical and Information Engineering, University of Witwatersrand, Johannesburg 2050, South Africa; maswat@pecopower.co.za; 2 Medical School, University of Nicosia, Nicosia 2421, Cyprus

**Keywords:** wrist extension, wrist flexion, electroencephalography (EEG), dynamometer, force

## Abstract

A brain–computer interface could control a bionic hand by interpreting electroencephalographic (EEG) signals associated with wrist extension (WE) and wrist flexion (WF) movements. Misinterpretations of the EEG may stem from variations in the force, speed and range of these movements. To address this, we designed, constructed and tested a novel dynamometer, the IsoReg, which regulates WE and WF movements during EEG recording experiments. The IsoReg restricts hand movements to isometric WE and WF, controlling their speed and range of motion. It measures movement force using a dual-load cell system that calculates the percentage of maximum voluntary contraction and displays it to help users control movement force. Linearity and measurement accuracy were tested, and the IsoReg’s performance was evaluated under typical EEG experimental conditions with 14 participants. The IsoReg demonstrated consistent linearity between applied and measured forces across the required force range, with a mean accuracy of 97% across all participants. The visual force gauge provided normalised force measurements with a mean accuracy exceeding 98.66% across all participants. All participants successfully controlled the motor tasks at the correct relative forces (with a mean accuracy of 89.90%) using the IsoReg, eliminating the impact of inherent force differences between typical WE and WF movements on the EEG analysis. The IsoReg offers a low-cost method for measuring and regulating movements in future neuromuscular studies, potentially leading to improved neural signal interpretation.

## 1. Introduction

Wrist extension (WE) and wrist flexion (WF) are crucial for stabilising, positioning and controlling the hand [1,2]; thereby enabling the performance of activities of daily living (ADLs). These ADLs include tasks such as using a knife, writing, turning a door handle or doorknob, opening a jar, donning pants, perineal cleansing and drinking from a cup [3,4]. A brain–computer interface (BCI) could empower individuals with hand motor impairments to regain the ability to perform a minimal set of ADLs that require WE and WF. For BCIs to be effective, it is essential to correctly interpret the user’s intention to perform WE and WF [5]. This necessitates the recording and processing of neural brain signals, such as electroencephalography (EEG), to elucidate and differentiate the signals associated with the unilateral control of WE and WF.

The typical forces and torques associated with WE differ from those associated with WF, as shown in Table 1. This discrepancy is partly due to the higher physiologic cross-sectional area of the wrist flexor muscles compared to the wrist extensor muscles [6,7]. A major challenge in EEG recording is the variation in the signals caused by changes in the force, the speed and the range of the finger, hand and arm movements, all of which can alter EEG signal patterns [8,9,10]. EEG recording experiments should thus incorporate devices and methods to evaluate and control the variation in these parameters. Isolating the EEG signal patterns associated with these parameters from those related to the kinematic differences between WE and WF could enhance EEG interpretation. Consequently, deeper insights could be gained on the neural control of real WE and WF and subsequently improve BCI performance. This could be applicable to BCI users who possess some residual hand movement capability, particularly those undergoing neuromuscular rehabilitation [11]. For BCI users without hand movement control, the neural activity from hand motor imagery can be extracted [11]. This study focussed on real wrist movements only, which enabled the movements to be regulated. Subsequent studies could explore the adaption of the analysis to imagined movements.

Previous EEG studies have used dynamometers or manipulandums to measure, regulate and display the forces of hand motor tasks (including hand grasps, wrist movements, pinch grips and shoulder abduction) while EEG was recorded in parallel [12,13,14,15,16,17,18]. These devices were not controlled by the recorded EEG. They provided real-time force feedback with customised graphical user interfaces that enabled participants to perform their respective motor tasks with targeted forces/torques, which were computed as a percentage of their respective maximum voluntary contractions (MVCs). Our objective was to develop a low-cost dynamometer that provides similar visual feedback capabilities, while ensuring that WE and WF movements were performed isometrically. This dynamometer would allow the regulation of the force, speed and range of motion of WE and WF movements during EEG recording experiments. The recorded EEG could be subsequently analysed offline with the aim of developing a BCI capable of controlling a bionic hand.

A summary of the studies that utilised dynamometers to measure the forces or torques associated with WE and WF is presented in Table 2. Two of the dynamometers met the requirements of our aim but were propriety and expensive [16,17]. The other dynamometers in Table 2 did not provide any visual feedback or were not associated with EEG experimentation.

Torque sensors (to measure torque of the movements about the wrist joint) and load cells (to measure the force applied due to movements) were used as transducers in the reviewed devices. The latter provides a cheaper alternative to the former. Hence, the design of our own low-cost dynamometer uses load cells to provide measurements of the force generated by WE and WF, perpendicular to the palm. Furthermore, a design based on load cells could be extended to enable force measurements of additional hand movements. This capability may make it suitable for a larger variety of EEG studies on hand movements.

This paper describes the design, construction and testing of our novel dynamometer, named the IsoReg. A key component of the testing evaluated the IsoReg’s correct functionality during an EEG recording experiment. The experiment required that the movement parameters of the WE and WF motor tasks be regulated in terms of force, speed and range of motion. This condition makes the recorded EEG data useful for a variety of studies that investigate the neural control signals associated with WE and WF motor tasks.

**Table 1:** Summary of the largest and smallest recorded wrist MVC forces from the literature. Reported torques (in Nm) were converted to forces (in N) using the reported lengths of the hands.

Reference	Largest MVC (N)		Smallest MVC (N)	
	WE	WF	WE	WF
[19]	68.88	88.27	27.37	35.20
[7]	28.57	56.12	18.37	25.51
[20]	40.82	61.22	27.93	33.52
[21]	63	73	45	54
[22]	214	-	-	-

**Table 2:** Summary of dynamometers used in the literature to measure wrist force or torque.

Motor Task for Which Force/Torque Was Measured	Type of Transducer	Used with EEG Recording	Visual Feedback	Reference
Isometric WE and WF torque performed at 15% of MVC	Strain gauge and commercial strain gauge amplifier	Yes	Yes	[16]
Peak isometric torque of WE and WF	Torque sensor (Scaime DF30-25 Nm)	No	Yes	[19]
WE and WF	Wristalyzer with strain gauge torque sensor	Yes	Yes	[17,23]
Peak isometric torque of WE and WF	Torque sensor (UTMII-20 Nm)	No	No	[7]
Grip force and isokinetic wrist torque	Miniature load cells (Honeywell Sensotec, 13/2244-06-10)	No	No	[20]
Isometric WE and WF in different positions	Le Bow load cells (136 kg capacity, Model No. 3397)	No	No	[21]
WE; flexion and extension of knee, elbow, neck and foot; shoulder and hip abduction; hip flexion.	Handheld dyno containing a modified Wika pressure gauge (0–300 N)	No	No	[22]

## 2. Materials and Methods

### 2.1. Design Specifications

**Figure 1:**
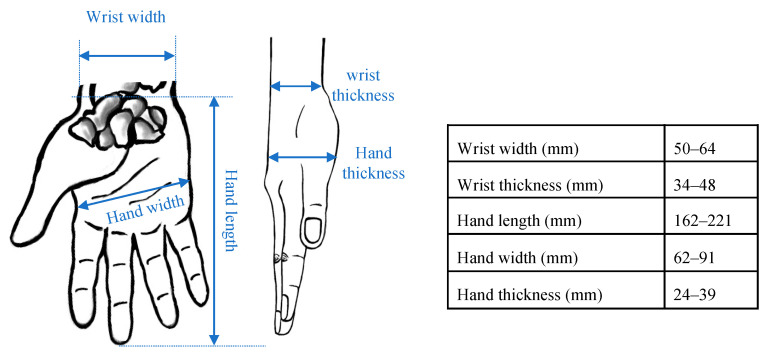
Typical dimensions of the human hand [30,31,32,33,34,35].

The design specifications are shown in Table 3. The IsoReg was designed considering our EEG experimental setup and assumptions, along with the range of expected forces as per Table 1. Our EEG experiment was similar to previous synchronous, visually cued sensorimotor BCI experiments [12,13,24,25,26,27]. EEG recordings were performed when participants were seated in a comfortable chair, facing a computer screen. The participants performed real WE and WF motor tasks, one hand at a time. Their motor tasks were regulated by the IsoReg during the recording of EEG, which did not control the IsoReg. Their hands were positioned midway between pronation and supination to ensure that gravity had an equal effect on WE and WF [16]. A force gauge displayed the relative force of the motor tasks on the computer screen [12,13,14,28]. The shoulders were kept level and shoulder abduction (for the arm performing in the motor tasks) was limited to 30° [29]. The duration of an EEG recording per participant did not exceed four hours to avoid mental and physical fatigue. The expected heights of the participants ranged between 1.9 and 1.5 m. The expected dimensions of the hands and wrists of the participants are shown in Figure 1.

### 2.2. Mechanical Design and Construction

**Figure 2:**
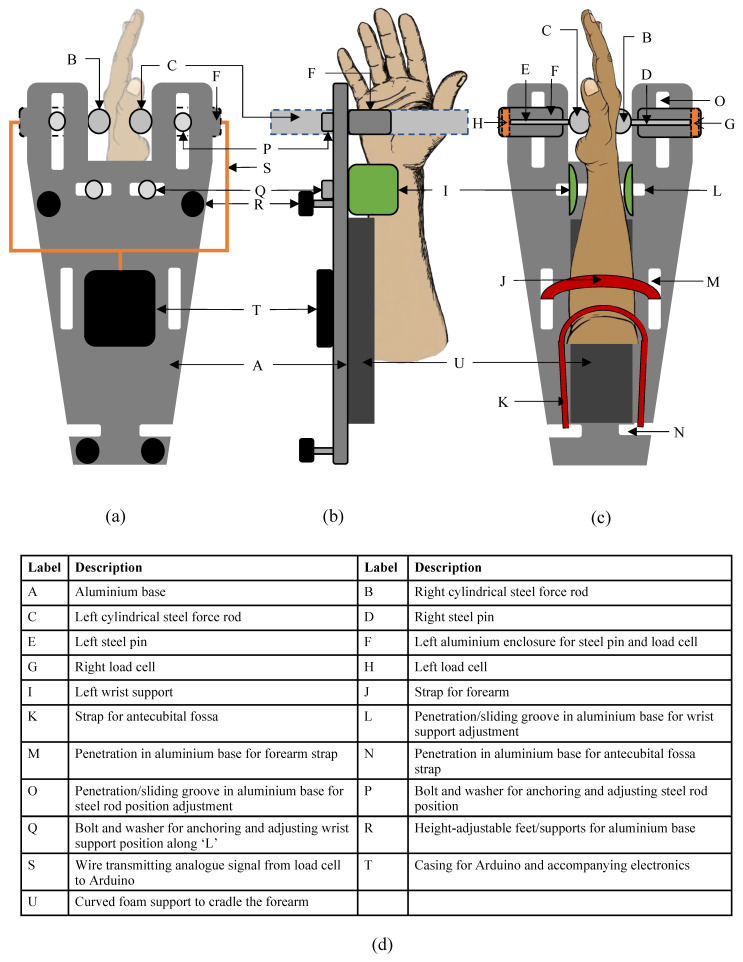
Major components and main measurements of the IsoReg shown from different views. (**a**) shows the top view, (**b**) shows the view of the left side and (**c**) shows the view from the underside/bottom of the IsoReg. (**d**) describes the labels A–U.

The main physical components of the IsoReg are shown in Figure 2. Appendix A contains photographs the IsoReg.

The base of the IsoReg was constructed with aluminium to meet the weight and strength specifications. Cylindrical force rods and wrist supports were used to keep the hand in place, thus isolating wrist movements to isometric WE and WF. The cylindrical rods held the hand in place midway along the length of the metacarpals. The wrist supports anchored the wrist in place just proximal to the wrist joint and at the distal end of the radius bone. This is shown in Figure 2. The wrist supports could slide laterally and medially to accommodate different wrists sizes. The positions of the force rods could be adjusted laterally, medially, distally and proximally to accommodate different hand sizes. (The anatomy of the hand and forearm as well as the different hand sizes and wrist sizes are shown in Figure 1). To reduce unwanted movements during the motor tasks, the forearm and elbow were held in place. The forearm was held down by a strap midway across the length of the middle of the forearm. The elbow was kept in place with a strap across the antecubital fossa. The straps were adjustable and attached to penetrations in the base plate. The support adjustments and straps are also shown in Figure 2.

The cylindrical steel force rods were connected to pins housed in aluminium cases that only allowed the pins to move medially and laterally. This design ensured that only forces perpendicular to the palm and metacarpals were measured. These pins were made of steel to prevent deformation and pushed onto the load cells. For a WE with the right hand (RH), the right steel force rod was pushed laterally and parallel to the metacarpals. This pushed the right steel pin onto the right load cell. Similarly, for a left-hand (LH) WF, the right half of the IsoReg was engaged, while for an LH WE and an RH WF, the left half of the IsoReg was engaged.

Thin pieces of firm foam covered the force rods, preventing pain in the palms and metacarpals during WF and WE, respectively. The same foam lined the wrist supports. These pieces of foam deformed minimally under the force conditions of this experiment. Thicker foam, glued to the aluminium base, cradled the forearm and helped to keep it in place. The foam additions (shown in the photos in Appendix A) made the IsoReg considerably more comfortable to use for the duration of the experiment.

### 2.3. Electronic Measurement System Design and Construction

**Figure 3:**
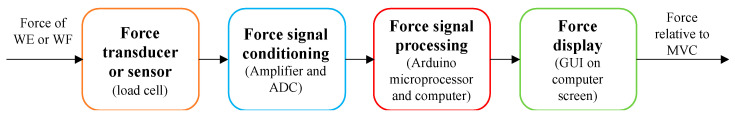
Block diagram of force measurement system.

The electronic measurement system of the IsoReg was designed and constructed using Bentley’s model, as shown in Figure 3 [36].

**Figure 4:**
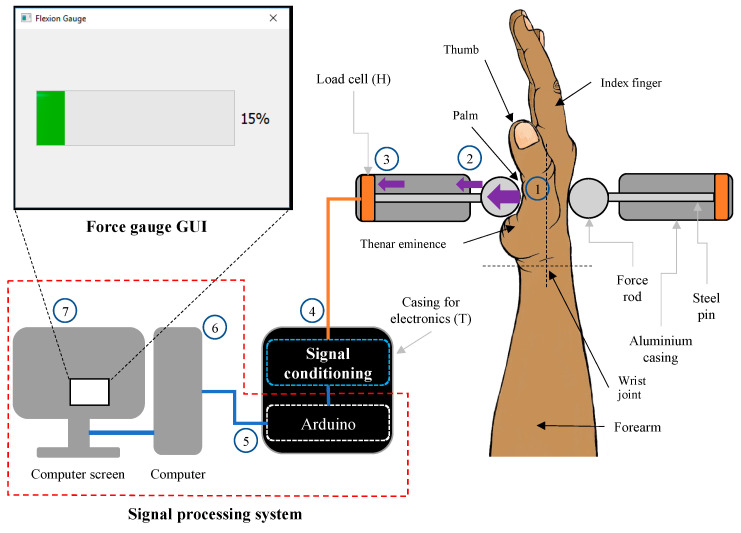
Demonstrating the mechanism of force measurement using RH WF as an example.

The basic mechanism of force measurement is shown in Figure 4. The circled numbers in the figure correspond to the following steps:

1. A participant performed WF with their RH. The hand tried to move anticlockwise about the wrist joint but was held in place by the wrist supports, forearm supports and left steel cylindrical rod. The palm of the hand pushed the left steel cylindrical force rod medially and perpendicular to the palm and metacarpals.
2. The force rod in turn pushed the left steel pin medially in its aluminium casing.
3. The left steel pin pushed onto the left load cell, which converted the force of the WF into a voltage.
4. This analogue voltage was then conditioned and converted to a digital force by the force conditioning system.
5. An Arduino Uno microprocessor computed the digital force signal and sent it to the computer.
6. The computer interpreted the force and computed the percentage of force relative to the stored MVC force value.
7. The force percentage was displayed on the force gauge interface on the computer screen.

The TE Connectivity FX1901 Compression Load Cell (Measurement Specialties (Europe), Ltd., Les Clayes-sous-Bois, France, part number FX1901-0001-0100-L) was selected as the force transducer, to meet the design specifications. (A similar load cell was previously used to measure pinch grips at 12% of MVC [37]). Our selected component provided a force measurement range of 0–445 N, contained a built-in Wheatstone bridge and exhibited negligible physical deflection when forces were applied. Furthermore, this component was small and light enough to fit into the steel rods, did not limit the bandwidth of the system, and its cost of $28 made it more economically suitable than commercial torque sensors.

**Figure 5:**
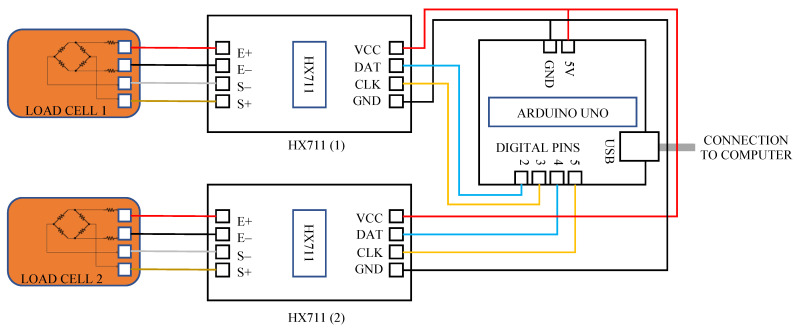
Circuit diagram of IsoReg electronics.

The analogue voltages transduced from the forces applied to load cells were conditioned prior to processing by the computer software. The conditioning was performed by two HX711 integrated chips (Avia Semiconductor (Xiamen Ltd., Xiamen, China) and an Arduino Uno microcontroller development board. This is shown by the circuit diagram in Figure 5. The Arduino, HX711 chips and the load cells were powered by 5 V supplied via the USB cable from the computer. The HX711 integrated chips included a 24-bit analogue-to-digital converter (ADC) and a built-in amplifier, making them ideal for weigh-scale applications [38]. The gains of the chips were set to 32 to amplify the analogue output voltage from the load cells (which ranged between 0 and 100 mV). The ADC provided a force resolution of 41.5 μN. The resolution is given by Equation (1), where *V_CC_* is the supply voltage from the Arduino Uno to the HX711 (5 V), *F_LCR_* is the range of forces of the load cell (445 N), *V_MA_* is the maximum analogue voltage from the load cell (100 mV) and *G* is the gain (32). The force resolution (*FR*) exceeded the required minimal force resolution (specified in Table 3).

(1) $$FR=\frac{V_{CC}\times F_{LCR}}{V_{MA}\times G\times 2^{24}}$$

Each HX711 chip provided a digital output voltage (between 0 and 5 V) to the Arduino. Data were sent serially from the Arduino to the computer via the USB port. The data transfer rate was controlled by the computer requesting data from the Arduino Uno. The software processes of the Arduino and computer are described in Section 2.4.

### 2.4. Software for Force Signal Processing

**Figure 6:**
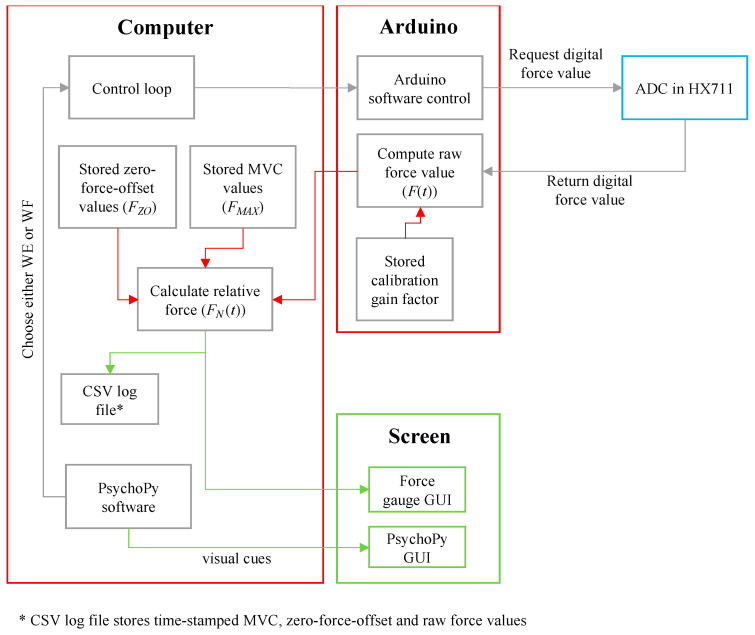
Third and main software routine to capture, calculate and display MVC-normalised real-time force data.

Three software routines were developed to collect the force data for one hand’s motor tasks. The routines are outlined below:

1. The first routine measured the resting force prior to the performance of the repetitive motor tasks. This resting force is denoted as the zero-force-offset value.
2. The second routine measured the MVC prior to the performance of the repetitive motor tasks.
3. The third routine measured, recorded and displayed the real-time normalised force data as a participant performed multiple repetitions of the motor tasks. This routine is portrayed in Figure 6. It is dependent on the first and second routines.

As shown in Figure 6, a customised PsychoPy2 software (Open Science Tools Ltd., Nottingham, England, v1.85.6) script [39] controlled the selection of motor tasks and the overall timing of the EEG recording experiment. Based on the selected motor task, the IsoReg displayed the force gauge for either WE or WF. For the selected motor task, the MVC-normalised force at a given sampled point in time (*F_N_*(*t*)), was calculated using Equation (2), where *F_MAX_* is the stored MVC value, *F_ZO_* is the stored zero-force-offset value and *F*(*t*) is the raw force value obtained from the Arduino.

(2) $$F_{N}(t)=\frac{{F(t)-F}_{ZO}}{F_{MAX}}$$

When the computer requested data from the Arduino for a specific motor task, the Arduino sent a raw force value (*F*(*t*)) back to the computer, which was then converted into an MVC-normalised force value (*F_N_*(*t*)). This loop was repeated every 90 ms, until the Psychopy software terminated the EEG recording session. A 90 ms refresh rate for the force gauges was sufficient to approximate the real-time force measurement display. The MVC, zero-force-offset values and time-stamped raw force values were saved to comma-separated-value (CSV) log files.

**Figure 7:**
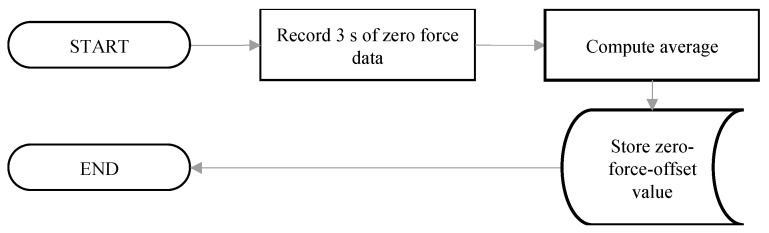
Second software routine for calculating the zero-force-offset values for each participant’s hand.

When the IsoReg was switched on, there were no external forces acting on the cylindrical force rods. When a participant inserted their hand between the force rods, forces were exerted onto the load cells while a participant’s hand was at rest. The first routine compensated for these forces [20]. While a participant kept their hand at rest, placed firmly between the force rods, the zero-force-offset value was calculated according to Figure 7. This was conducted for both load cells for both hands of each participant.

**Figure 8:**
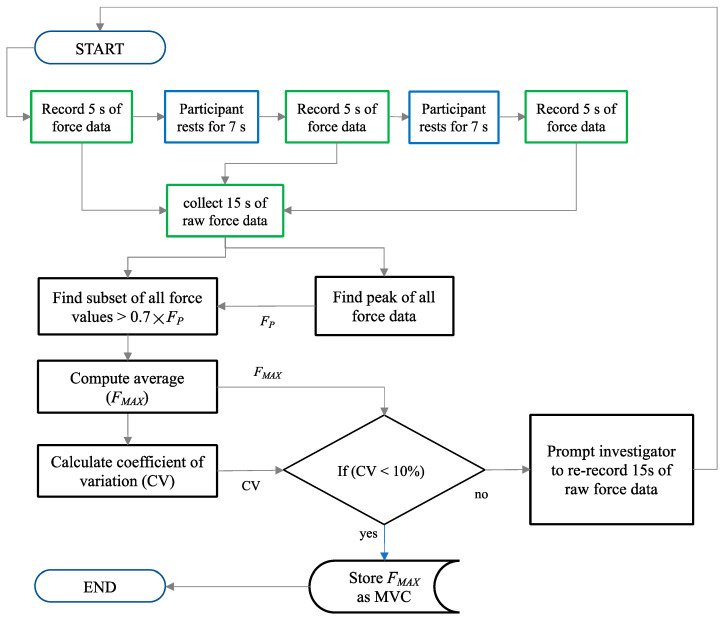
First software routine for calculating the WE and WF MVCs for each participant’s hand.

Figure 8 shows the software routine that controlled the testing of the MVC measurements for each hand and for each motor task (detailed in Section 2.6). The coefficient of variation (CV) was used to ensure that the participants performed their respective maximum contractions consistently across three periods of MVC recording [40]. A CV lower than 10% was indicated as effective in previous studies [19]. The CV was calculated using Equation (3) by first isolating a subset of all force values greater than 0.7 × *F_P_* (denoted as *F*_0.7*P*_). *F_P_* was the peak force value from all 15 s of recorded force data, as illustrated in Figure 8.

(3) $$CV=\frac{standard\;deviation\;\left\{F_{0.7P}\right\}}{mean\;\left\{F_{0.7P}\right\}}$$

*F_MAX_* was calculated as the mean of *F*_0.7*P*_, as shown in Figure 8. This value minimised the risk of capturing a noisy or erroneous spike value as the MVC [20]. Hence, the MVC was calculated using only the upper range of values from the 15 s of data recorded during the performance of WE or WF at full strength.

### 2.5. Calibration

For calibration, the load cells were temporarily removed from their aluminium enclosures. A 3 kg metal mass was applied to the centre of each of the load cells while they were positioned on a flat surface. A calibration gain variable was iteratively adjusted until the force measured by the Arduino matched the applied force of the metal mass. The final value of the calibration gain factor was set to 28.

The load cells were then reinstalled in their aluminium enclosures. A portable hanging scale (Marsden Weighing Group, Rotherham, United Kingdom, part number TC/OCS-L-30) was subsequentially used to manually apply and measure varying horizontal forces to the cylindrical steel force rods. The measured forces of the Arduino were compared to the forces of the portable hanging scale to verify that the calibration gain variable was correctly set. Calibration was performed once, prior to testing in the EEG recording experiment.

### 2.6. Testing Protocol

The IsoReg was tested through three consecutive steps:

1. Test 1: The accuracy of the measured raw force values was tested. This test was the starting point of the three signal processing routines.
2. Test 2: Once the basic raw force measurement was verified, the calculation and display of the relative forces were tested. This verified that users of the device received sufficiently accurate force feedback.
3. Test 3: Subsequently, the device was tested within an EEG recording experiment. The force data during this experiment was logged and analysed to verify that participants did in fact perform MVC-normalised repetitions of WE and WF.

**Figure 9:**
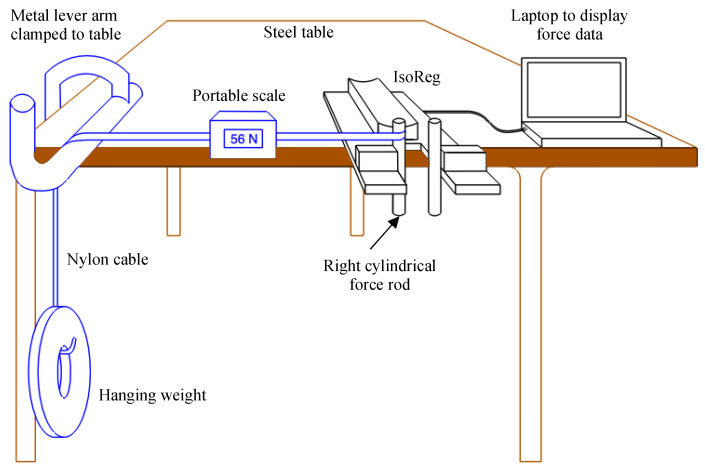
The experimental setup for Test 1 and Test 2 for the right cylindrical rod. The left cylindrical rod was tested in a similar manner.

Test 1 and Test 2 were implemented using the setup shown in Figure 9. The MVCs for WE and WF were manually set to 100 N. The applied force (*AF*) was varied over the range of expected forces (0–214 N) by varying the mass of hanging weights. The applied force was measured on the tension of the nylon cable using a portable hanging scale (part number TC/OCS-L-30) with a range of 0–300 N. Before each weight was applied, the zero-force-offset values registered by the IsoReg were recorded. The varying forces were applied to the left and right cylindrical rods and the corresponding raw and MVC-normalised force values (measured and computed by the IsoReg, respectively) were recorded.

In Test 1, the errors between the applied force (*AF*) and the raw force values measured by the IsoReg (*F*) were calculated using Equation (4).

(4) $$Error1=\frac{\left|AF-F\right|}{AF}$$

In Test 2, the MVC-normalised applied forces were calculated manually using Equation (2). These values are denoted as *AF_N_.* The errors between the manually calculated MVC-normalised forces values (*AF_N_*) and the MVC-normalised forces displayed on the force gauges (*F_N_*) were calculated using Equation (5).

(5) $$Error2=\frac{\left|{AF}_{N}-F_{N}\right|}{{AF}_{N}}$$

Test 3 involved 14 participants, who were right-handed, healthy, untrained, student volunteers, without prior wrist injuries, between 20 and 30 years old. Eight participants were male and six were female. They all completed an online handedness questionnaire, adapted from [41], to verify that they were right-handed [42]. Their participation followed ethics approval from the Medical Human Research Ethics Committee at the University of Witwatersrand (clearance certificate number M190607).

**Figure 10:**
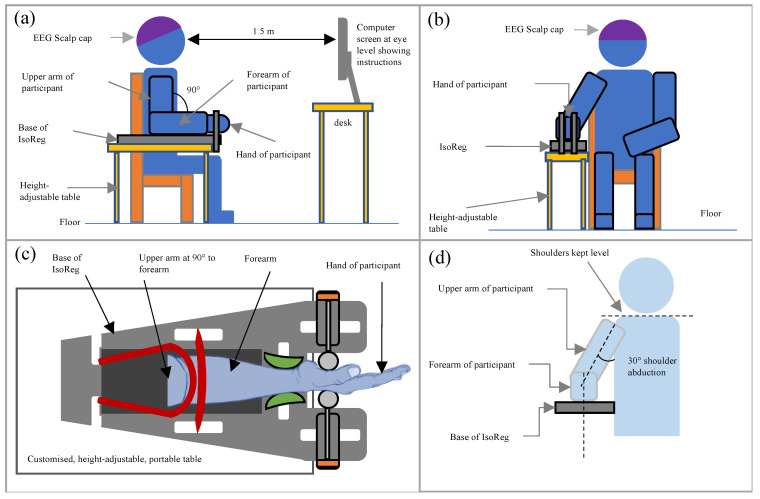
Depictions of how a participant was seated in the lab with all the surrounding equipment for EEG recording. (**a**) shows the view from the side. (**b**) shows the view from the front. (**c**) shows the top view of the hand strapped to the base of the IsoReg. (**d**) shows the front view of a seated participant, showing the position of the shoulders and upper arm.

Figure 10 illustrates the experimental configuration for Test 3, which is an example of typical EEG recording experiments. As shown, a participant sat in a comfortable chair, facing the computer screen, with one arm strapped into the IsoReg, which was fixed to its portable table. The participant’s forearm was positioned facing directly towards the computer screen. The hand and forearm were positioned midway between pronation and supination so that gravity had an equal effect on WE and WF [16].

Each participant was trained on how to perform the motor tasks correctly with the first hand secured in the IsoReg. The zero-force-offset value as well as the MVCs for both WE and WF on one hand were measured and recorded. (This is described in the next paragraph.) Thereafter, the participant followed visual instructions (controlled by the Psychopy software script and displayed on a computer screen) to perform repetitions of the motor tasks (detailed in the subsequent paragraph). Meanwhile, two processes were run in parallel: (1) EEG data were recorded from 128 electrodes, and (2) the IsoReg simultaneously computed and recorded the instantaneous MVC-normalised forces (*F_N_*) of the motor tasks, which were displayed using the force gauge on the computer screen. The participant rested for 20 min, and the process was repeated for the other hand. The EEG data were recorded from 128 active Ag/AgCl electrodes, positioned using the 10-5 system in an actiCAP (Brain Products GmbH, Gilching, Germany).

To measure the MVCs, the resting forces (zero-force-offset values) for both hands were first measured and recorded). Thereafter, for each hand and for each motor task, the participant followed the visual instructions shown on the computer screen. These instructions were prompted by the IsoReg software (version 2) to measure and record the MVC according to the procedure outlined below:

- The participant rested for seven seconds [34]. For the last four seconds of this rest period, a countdown timer prepared the participant for movement.
- Upon instruction to contract, the participant performed and sustained a WE or WF movement with all the strength of their wrist for five seconds [37] until instructed to rest again.
- Steps 1–3 were repeated three times.

Participants were instructed to focus on movement about the wrist joint only and were verbally encouraged to exert maximum effort during each contraction [43]. If the recorded forces from the three repetitions of MVC measurements were not consistent, the three repetitions for MVC measurement were repeated.

**Figure 11:**
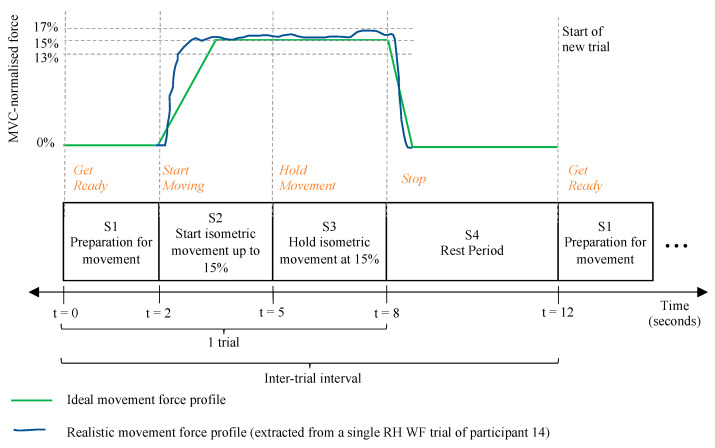
Timing diagram of a single trial and the related changes in MVC-normalised wrist force. Visual cues are shown in orange text. During S2, the participant tried to obtain the relative force of either WE or WF up to 15% as fast as possible. During S3, the participant tried to sustain the isometric movement at 15%, but maintaining a relative force between 13% and 17% was deemed acceptable.

The two motor tasks consisted of controlled, MVC-normalised, visually cued, sustained isometric versions of WE and WF. The motor tasks were performed for each hand in batches of 20 repetitions each. Batches of WE and WF were performed in alternating order, but the recording sessions for each hand always started with a batch of WE. Each WE or WF repetition formed an 8 s trial and was made up of three segments (S1–S3). This was followed by a 4 s rest period (S4). The inter-trial interval was 12 s long. The segments of a single trial and its rest period are shown in Figure 11. The timed instructions for S1–S4 were coded and displayed alongside the force gauge of the IsoReg on the same computer screen. The total data recording procedure lasted approximately three hours for each participant. When performing the repetitions of WE and WF, participants were trained to keep the elbow, shoulder and forearm in a fixed position, with the hand positioned in the IsoReg as per Figure 10. They had to avoid curling the fingers to try close the hand during WF and avoid stretching the fingers backwards during WE. They were also instructed to concentrate on the correct performance of the motor tasks and avoid other cognitive tasks.

**Figure 12:**
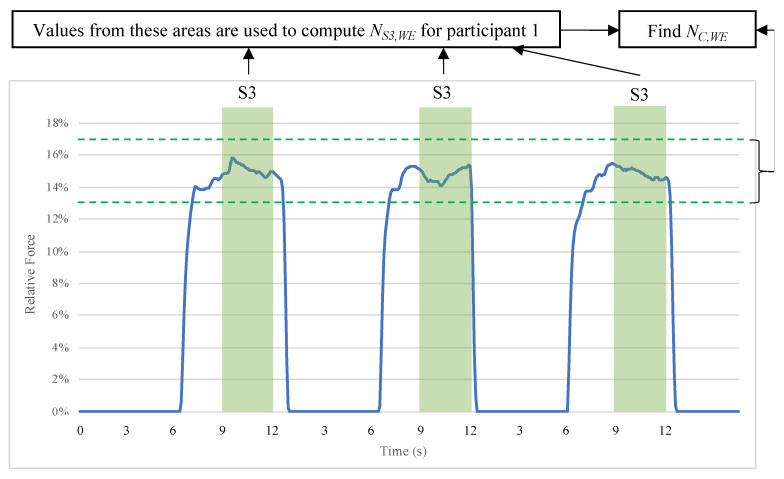
Testing method applied to determine the degree of force normalisation. *F_N_*(*t*) for participant 1 for three repetitions of WE with the RH is shown as an example (blue line). Ideally, all *F_N_* values during the periods of S3 (green strips) should lie between the dotted green lines. This method was used for all RH WE and WF repetitions and for all LH WE and WF repetitions.

The time-stamped, logged force values for all repetitions of WE and WF were analysed per participant. All *F_N_* values that the IsoReg measured during the S3 (sustained movement) period were extracted. These values were used to determine whether a participant performed the WE and WF motor tasks at their correct respective forces (13–17%). This is shown graphically in Figure 12. The absolute value of the difference between the WE and WF accuracies of force normalisation (DNF) was calculated using Equation (6).

(6) $$DNF=\left|\frac{N_{C,WE}}{N_{S3,WE}}-\frac{N_{C,WF}}{N_{S3,WF}}\right|$$

*N*_*S*3,*WE*_ denotes the total number of *F_N_* values logged during the S3 portion of WE trials.

*N*_*C*,*WE*_ denotes the total number of *F_N_* values logged during the S3 portion of WE trials that have values between 0.13 and 0.17, i.e., {0.13 < *F_N_* < 0.17}.

*N_S3,WF_* denotes the total number of *F_N_* values logged during the S3 portion of WF trials.

*N_C,WF_* denotes the total number of *F_N_* values logged during the S3 portion of WF trials, where {0.13 < *F_N_* < 0.17}.

## 3. Results

The measured and calculated forces and their corresponding average measurement errors in Tests 1 and 2 of the left and right cylindrical rods are shown in Table 4 and Table 5, respectively. For Test 1, the mean errors calculated for the testing of the left and right cylindrical rods were 1.73% and 2.79%, respectively. For Test 2, the mean errors calculated for the left and right cylindrical rods results were 0.87% and 1.34%, respectively.

**Figure 13:**
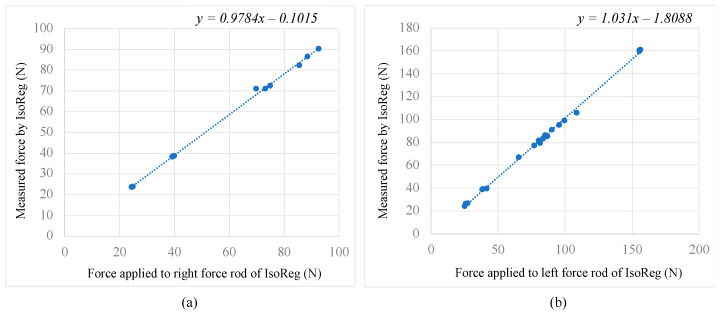
The measured forces vs. the force applied for the right cylindrical force rod (**a**) and the left cylindrical force rod (**b**).

Figure 13 displays a scatter plot and linear regression of the applied force (*AF*) and the measured raw force (*F*) values in Table 4 and Table 5. A linear relationship between *AF* and *F* can be observed for both right (Figure 13a, Table 4) and left rods (Figure 13b, Table 5). The ideal formula for the trendlines is *y* = *x*, which is close to the formula of the trendlines of the plots. Hence, the force applied to the cylindrical force rods was directly proportional to the force measured by the IsoReg.

The results from Test 3 (accuracy values of WE and WF force normalisation and the DNF results) for the RH and LH are shown in Table 6 and Table 7, respectively. The mean force normalisation accuracies across all participants for RH WE, RH WF, LH WE and LH WF were 89.97%, 90.30%, 88.75% and 90.58%. The overall mean force normalisation accuracy across Table 6 and Table 7 (considering values for all participants, both hands and both motor tasks) was 89.90% (SD 9.22%). The mean DNF values across all participants for the RH (Table 6) and LH (Table 7) were 3.81% (SD 3.64%) and 4.79% (SD 3.78%), respectively. The overall mean DNF across both tables was 4.30% (SD 3.66%).

## 4. Discussion

The interpretation of the neural control of wrist extension (WE) and wrist flexion (WF) movements in EEG recording experiments can be improved using a dynamometer, with real-time visual force feedback. Firstly, the dynamometer can normalise the inherent force differences between WE and WF [19]. Secondly, it can regulate the speed and range of motion—by fixing the position of the hand and forearm—thereby limiting WE and WF to isometric movements. Our dynamometer, the IsoReg, was designed and constructed to meet these goals in accordance with the specifications described in Table 3. This made it affordable, portable and comfortable and enabled participants to view and log the real-time forces of their WE and WF movements relative to their respective MVCs. The performance accuracies of the IsoReg were tested. These are discussed in the next two paragraphs.

The mean measurement accuracy values of Test 1 and Test 2 exceeded 97%, meeting the minimum design accuracy specification of 96%. Hence, the IsoReg was deemed sufficiently accurate in measuring raw force values and displaying normalised force values to participants. Furthermore, the forces applied to the cylindrical force rods were directly proportional to the forces measured by the IsoReg. Some commercial dynamometers are capable of transducing forces or torques of hand movements with similar accuracy values of 98% [44]. The corresponding accuracies of the devices reviewed in Table 2 were not reported; hence, they could not be compared to the IsoReg. Instead, these devices were used to analyse and report on the force characteristics of different types of WE and WF motor tasks. In comparison, our study is advantageous since the accuracy of the IsoReg was validated prior to the analysis of its force measurements.

The accuracy values of force normalisation were not reported in previous EEG studies utilising hand movement dynamometers or manipulandums [12,13,14,15,17,18,45,46]. A benchmark for the IsoReg’s accuracies of force normalisation could be found in a handgrip neuromuscular study that relied on functional magnetic resonance imaging (fMRI) [47]. This former study calculated root-mean-square errors between the target force trajectory and each participant’s force trajectory, for each trial and each hand, similar to the IsoReg error analysis in Test 3. The study reported an overall mean accuracy of 96.5% (SD 1%) across all trials, both motor tasks and both hands. The IsoReg’s mean accuracy in Test 3 was 89.90% (SD 9.22%). This lower performance may stem from a few comparative differences. Their study involved a commercial grade dynamometer, hand grasp motor tasks (which may be easier to control) and fewer unilateral trials per participant (42 vs. our 200). The IsoReg’s Test 3 results did demonstrate, however, that the IsoReg enabled the normalisation of 89.90% of all wrist forces (measured during the S3 segments of the trials). We thus deemed the normalisation as sufficient. The DNF results from Test 3 further indicated that the normalisation of WE and WF movements were similar. The comparative handgrip-fMRI study did not compute a metric similar to the DNF. Instead, it compared the level of normalisation between motor tasks by calculating the difference between their respective mean force normalisation accuracies (1.5%). Corresponding values for the IsoReg were calculated at 0.33% for the RH (difference between 89.97% WE and 90.30% WF mean accuracies in Table 6) and 1.83% for the LH (difference between 88.75% WE and 90.58% WF mean accuracies in Table 7). With an average value of 1.08% averaged across both hands, the IsoReg performed similarly to the dynamometer used in the fMRI study in terms of equalising the forces between two motor tasks.

The combined results of Test 1, Test 2 and Test 3 implied that any differences in the WE and WF neural control signals extracted from recorded EEG were not due to the inherent force differences associated with typical WE and WF movements. As discussed above, these tests were limited in previous EEG and force studies involving dynamometers to measure the forces of WE, WF and other hand movements. Applying these tests to future EEG studies interpreting real hand movement control could lead to improved EEG signal interpretation.

Dynamometers have not been prevalently used in EEG-based BCI studies, despite their advantage in regulating movement parameters. This may be partially due to many EEG-based BCI studies investigating the neural control of hand motor imagery instead of real hand movements [48]. Only two studies were found that used dynamometers to regulate WE and WF motor tasks while EEG was recorded [16]. However, these studies were not BCI studies. Our study was thus novel, since it used a dynamometer (the IsoReg) to measure and regulate isometric WE and WF repetitions, with the aim of improving the EEG-based BCI interpretation of these movements. Only three other BCI studies, employing a dynamometer and aiming to improve EEG interpretation, were found [18,45,46]. These studies used a handgrip dynamometer in a series of experiments to measure and control the speed and force of real palmar, lateral and pinch grasps. One of these studies used the dynamometer to regulate the speed and force of these grasps to isolate these parameters from signal patterns associated with the kinematic differences in the grasps [46], similar to the approach used in our study. In the other two studies, the dynamometer was used to investigate changes in EEG signal patterns resulting from the differences in the speed and force of the grasps [18,45].

The IsoReg could be used in future EEG recording experiments (similar to the latter two studies) to investigate the neural signal pattern changes associated with different speeds and forces of WE and WF. Thereafter, the IsoReg could be adjusted mechanically to elucidate the neural control of finger extension and flexion, using an experiment similar to Test 3. The adjustments could involve shifting the cylindrical steel rods, load cells and their enclosures distally to hold the fingers in place and adding supports to secure the palm and metacarpals. The steel rods could then be positioned between the distal and middle phalanges and transduce forces from the fingers laterally to the load cells. No changes to the electronics and software would be required. The IsoReg is limited to measuring isometric WE and WF. To enable force measurement during concentric and eccentric movements, the IsoReg could be redesigned to utilise a cable and pulley system with load cells specialised for measuring cable tension. This, however, may incur additional costs.

Another limitation of the IsoReg is its current inability to hold the fingers in place. In this study, healthy participants were able to avoid the extension and flexion of their fingers as they performed isometric WE and WF repetitions. However, patients presenting with finger spasticity or other neuromuscular impairments may lack sufficient control of their fingers. Hence, for future EEG experiments with the IsoReg, particularly involving these motor-impaired individuals, additional supporting steel rods should be added to the IsoReg to prevent involuntary finger movements.

WE and WF are among the first movements that these individuals relearn during rehabilitation [2]. The IsoReg, or variations thereof, could, in theory, be used as part of the neurorehabilitation therapy of motor-impaired individuals, particularly stroke victims [49]. The IsoReg is currently not controlled by an EEG-based BCI. With only the adjustments described in the previous paragraph, it could be used to track the forces of isometric WE and WF as a marker of improvement throughout the course of conventional occupational therapy sessions. This would be applicable to stroke patients possessing some hand movement control. Further modifications to the IsoReg could convert it into a robotic orthotic device controlled by an EEG-based BCI. This orthotic device could be used by stroke patients with limited to no hand motor control as part of BCI therapy to enhance neuroplasticity [49]. Patients will be trained to enhance their motor imagery that would control the orthotic device to push the hand to perform WE and WF, while it simultaneously measures the forces applied to the hand. This requires the addition of motors to IsoReg and enabling their control by a BCI that interprets WE and WF motor imagery patterns extracted from EEG.

The EEG signal processing techniques necessary to interpret the WE and WF control signal patterns are not presented in this article. The focus of this article is the design and evaluation of the IsoReg to aid EEG recording processes, which are explained in Section 2.1 and Section 2.6. The recorded EEG data were processed using techniques similar to those used in our previous studies [5,50,51]. These techniques included preprocessing and artifact removal; source localisation using independent component analysis; time–frequency feature extraction; feature selection using the Bhattacharya distance; and classification using Mahalanobis distance clustering. The combination of these techniques could be used, in the future, to characterise the differences in signal patterns associated with varied forces and speeds of WE and WF. This would require the recording of additional EEG data, where the IsoReg will be used to vary the speeds and forces of WE and WF motors and not regulate them, as conducted in this study.

The aforementioned possible modifications to the IsoReg would require careful redesign to maintain its low-cost nature. Its construction cost of $111 USD, in comparison to the costs exceeding $5000 USD in three of the seven devices listed in Table 2, makes it accessible to researchers. The low-cost design of the IsoReg could be leveraged by researchers to construct their own affordable devices for use in future BCI or neuromuscular studies. These studies may include movements such as finger flexion and extension, and flexion and extension about the elbow, shoulder, knee, ankle and wrist. An increased prevalence in the use of affordable devices for regulating and measuring movement parameters may lead to improved sensorimotor signal analysis.

## Figures and Tables

**Table 3:** Design specifications of the IsoReg and its support table.

Design Consideration	Specification
Construction	Adjust to accommodate typical dimension of human hands, forearms and wrists for both right and left hands. Comfortable usage for up to four hours.
Maximum mass	3 kg (for portability)
Dimensions	<600 mm (length); <350 mm (width)
Force range	0–214 N (from Table 1)
Resolution	1% of minimum MVC from Table 1 = 0.28 N
Frequency range of input signals to handle	0–100 Hz
Accuracy	>96%
Output display	Force gauge on computer screen with a range of 0–100% relative to MVC
Output data logging	Time-stamped forces saved in CSV file
Dimensions of supporting table	<600 mm (length); <350 mm (width), 500–800 mm (adjustable height); easy assembly for portability
Maximum mass of supporting table	10 kg for portability
Expected force range applied to supporting table	0–214 N (from Table 1)
Cost	<$140 USD

**Table 4:** Measured forces and average measurement errors in the left cylindrical rods, for Test 1 and Test 2. *AF* denotes the applied force, *F* the steady-state raw force from the IsoReg, *F_ZO_* the zero-force-offset value, *F_N_* the steady-state normalised force and *AF_N_* the manually calculated MVC-normalised force. When *AF* exceeded 100 N (the MVC value), the error for *F_N_* could not be calculated since the force gauge was saturated at 99%.

	Test 1		Test 2			
*AF* (N)	*F* (N)	Error1 for *F* (%)	*F_ZO_* (*N*)	*F_N_* on Gauge (N)	*AF_N_* (N)	Error2 for *F_N_* (%)
24.80	24.28	2.10	0.06	24	24.22	0.91
25.63	26.24	2.38	0.06	26	26.18	0.68
27	26.97	0.11	0.06	26	26.91	3.38
41.10	39.85	3.04	−0.07	39	39.92	2.30
38.30	39.04	1.93	−0.07	39	39.11	0.28
38.17	38.81	1.67	−0.07	38	38.88	2.26
65.31	67.02	2.62	0.16	67	66.86	0.21
76.83	77.27	0.57	0.16	76	77.11	1.44
86.53	85.44	1.26	0.16	85	85.28	0.33
84.88	86.43	1.83	0.16	86	86.27	0.32
80	81.57	1.96	0.16	81	81.41	0.51
83.49	83.11	0.45	0.16	83	82.96	0.05
84.28	84.56	0.33	−0.05	84	84.61	0.72
90.06	91.04	1.08	−0.05	91	91.09	0.10
81.34	79.55	2.20	−0.05	79	79.60	0.75
99.30	98.97	0.33	−0.28	99	99.25	0.26
108.30	105.85	2.26	−0.28	99	106.13	N/A
95.40	95.03	0.39	−0.28	95	95.31	0.33
156	161	3.21	0.45	99	160.55	N/A
155.14	160.70	3.58	0.45	99	160.25	N/A
155.10	159.90	3.09	0.45	99	159.45	N/A
Mean		1.73				0.87

**Table 5:** Measured forces and average measurement errors in the right cylindrical rods, for Test 1 and Test 2. *AF* denotes the applied force, *F* the steady-state raw force from the IsoReg, *F_ZO_* the zero-force-offset value, *F_N_* the steady-state normalised force and *AF_N_* the manually calculated MVC-normalised force.

	Test 1		Test 2			
*AF* (N)	*F* (N)	Error1 for *F* (%)	*F_ZO_* (*N*)	*F_N_* on Gauge (N)	*AF_N_* (N)	Error2 for *F_N_* (%)
39.25	38.18	2.74	0.17	38	38.01	0.02
39.35	38.38	2.48	0.17	38	38.21	0.54
39.83	38.65	2.96	0.17	38	38.48	1.26
24.45	23.66	3.22	1.20	22	22.46	2.06
24.30	23.67	2.58	1.20	22	22.47	2.10
24.84	24.04	3.20	1.20	22	22.84	3.69
73.20	71.12	2.84	1.73	69	69.39	0.56
74.90	72.49	3.21	1.73	69	70.76	2.49
69.70	71.12	2.04	1.73	70	69.39	0.88
88.50	86.56	2.19	1.75	84	84.81	0.96
92.60	90.35	2.43	1.75	88	88.6	0.68
85.50	82.39	3.64	1.75	80	80.64	0.79
Mean		2.79				1.34

**Table 6:** Test 3 results for wrist movement repetitions with the RH. N/A denotes where force data was not available.

Participant Number	Accuracy of Force Normalisation for WE $\left(\frac{N_{C,WE}}{N_{S3,WE}}\right)$ (%)	Accuracy of Force Normalisation for WF $\left(\frac{N_{C,WF}}{N_{S3,WF}}\right)$ (%)	DNF (%)
1	92.37	89.21	3.16
2	97.83	95.93	1.9
3	95.08	99.81	4.73
4	53.54	65.09	11.55
5	96.23	97.27	1.04
6	82.86	72.38	10.48
7	96.5	92.76	3.74
8	88.10	92.57	4.47
9	98.67	99.35	0.68
10	86.97	85.35	1.62
11	96.31	97.64	1.34
12	95.14	96.21	1.07
13	N/A	N/A	N/A
14	N/A	N/A	N/A
Mean (SD)	89.97 (12.46)	90.3 (11.02)	3.81 (3.64)

**Table 7:** Test 3 results for wrist movement repetitions with the LH. N/A denotes where force data was not available.

Participant Number	Accuracy of Force Normalisation for WE $\left(\frac{N_{C,WE}}{N_{S3,WE}}\right)$ (%)	Accuracy of Force Normalisation for WF $\left(\frac{N_{C,WF}}{N_{S3,WF}}\right)$	DNF (%)
1	93.35	94.33	0.98
2	89.86	95.83	5.97
3	89.96	94.75	4.79
4	81.92	81.58	0.33
5	83.83	88.47	4.64
6	92.86	88.38	4.48
7	93	94.81	1.81
8	83.38	70.47	12.91
9	94.92	98.96	4.04
10	81.47	92.65	11.18
11	91.72	94.94	3.22
12	88.74	91.81	3.06
13	N/A	N/A	N/A
14	N/A	N/A	N/A
Mean (SD)	88.75 (4.85)	90.58 (7.79)	4.79 (3.78)

**Figure A1:**
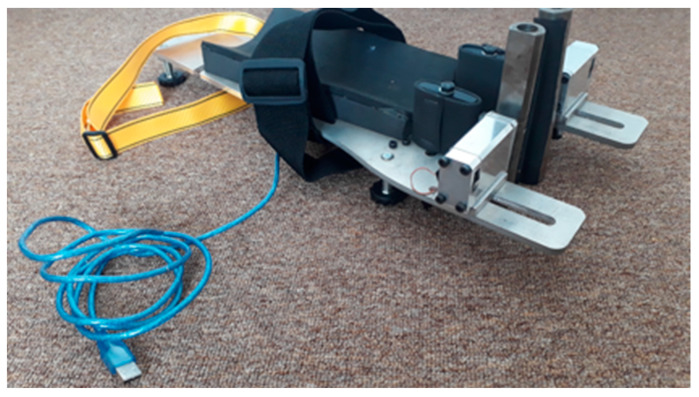
Picture 1 of IsoReg.

**Figure A2:**
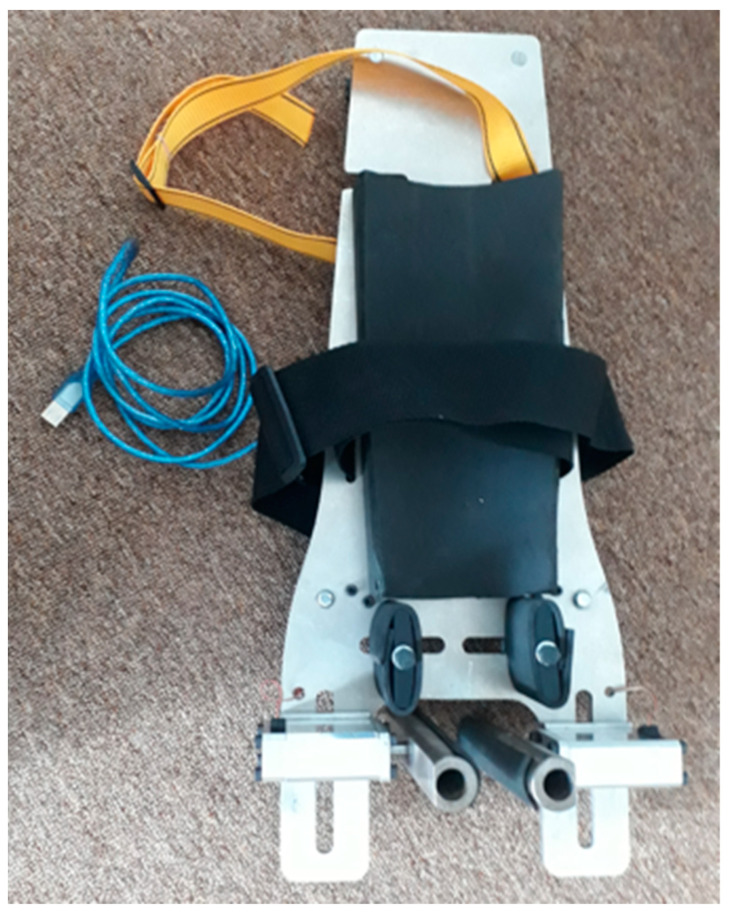
Picture 2 of IsoReg.

## Data Availability

Force and EEG data are unavailable due to privacy or ethical restrictions.
